# Dual anti-HER2 therapy combined with chemotherapy as a novel neoadjuvant treatment option for locally advanced HER2-positive and microsatellite stable colon cancer

**DOI:** 10.1093/pcmedi/pbae033

**Published:** 2024-12-10

**Authors:** Huayang Pang, Xiufeng Chen, Menghua Yan, Hao Sun

**Affiliations:** Department of Gastrointestinal Cancer Center, Chongqing University Cancer Hospital, Chongqing 400030, China; Department of Gastrointestinal Cancer Center, Chongqing University Cancer Hospital, Chongqing 400030, China; Department of Gastrointestinal Cancer Center, Chongqing University Cancer Hospital, Chongqing 400030, China; Department of Gastrointestinal Cancer Center, Chongqing University Cancer Hospital, Chongqing 400030, China

Dear Editor,

Over the past decade, multiple clinical trials have demonstrated the promising efficacy of anti-HER2 regimens in refractory HER2-positive metastatic colorectal cancer patients [1, 2]. Based on this compelling evidence, the current national comprehensive cancer network (NCCN) guidelines have incorporated anti-HER2 therapy (trastuzumab plus lapatinib, trastuzumab plus pertuzumab, trastuzumab plus tucatinib, and T-DXd) as a late-line treatment option for these individuals [3]. Therefore, the further evaluation of HER2-targeted therapy in neoadjuvant treatment holds significant clinical implications for expanding its benefits to a wider patient population, especially for those with microsatellite stable (MSS) status. We have previously reported the first case of a patient with resectable metastatic HER2-positive and MSS colon cancer who achieved a pathologic complete response (pCR) to neoadjuvant chemotherapy combined with dual anti-HER2 treatment, namely trastuzumab and pertuzumab [4]. Here, we further present the successful application of this novel neoadjuvant therapy to a locally advanced colon cancer patient with HER2-positive and MSS status.

A 69-year-old male patient presented to our department with acute periumbilical abdominal pain persisting for 4 days. This patient had recently undergone colonoscopy at another medical institution, which revealed a space-occupying lesion in the ascending colon. Additionally, the patient reported a history of nasopharyngeal carcinoma spanning two decades and comorbidities including hypertension and diabetes mellitus for 10 years. After admission, laboratory tests demonstrated normal levels of tumor markers. Contrast-enhanced computed tomography (CT) imaging of the chest, abdomen, and pelvis showed thickening of the proximal wall of the ascending colon invading the duodenum along with several adjacent small lymph nodes (cT4bN + M0, Fig. 1A). Colonoscopy confirmed adenocarcinoma at the hepatic flexure with stenosis (Fig. 1B). Furthermore, gastroscopy unexpectedly identified an early adenocarcinoma located in the gastric body (cT1N0M0, Fig. 1C). Subsequent immunohistochemical (IHC) analysis revealed that colon adenocarcinomas exhibited MSS status as well as negative HER2 expression (IHC 0 score, Fig. S1A, see online supplementary material). Similarly, the IHC results of gastric cancer were consistent with those of colon cancer, showing MSS status and an IHC score of 1. Several days later, next-generation sequencing (NGS) of biopsy tissue from colon cancer showed ERBB2 gene amplification (Fig. S1B), accompanied by an exon 20 insertion mutation [variant allele frequency (VAF) 46.51%]. Furthermore, the NGS analysis identified APC exon 16 mutation (VAF 15.18%), ARID1A exon 10 mutation (VAF 12.53%), TP53 exon 6 mutation (VAF 14.67%), as well as MSS and no mutations in KRAS, NRAS, BRAF, and PIK3CA.

**Figure 1.**
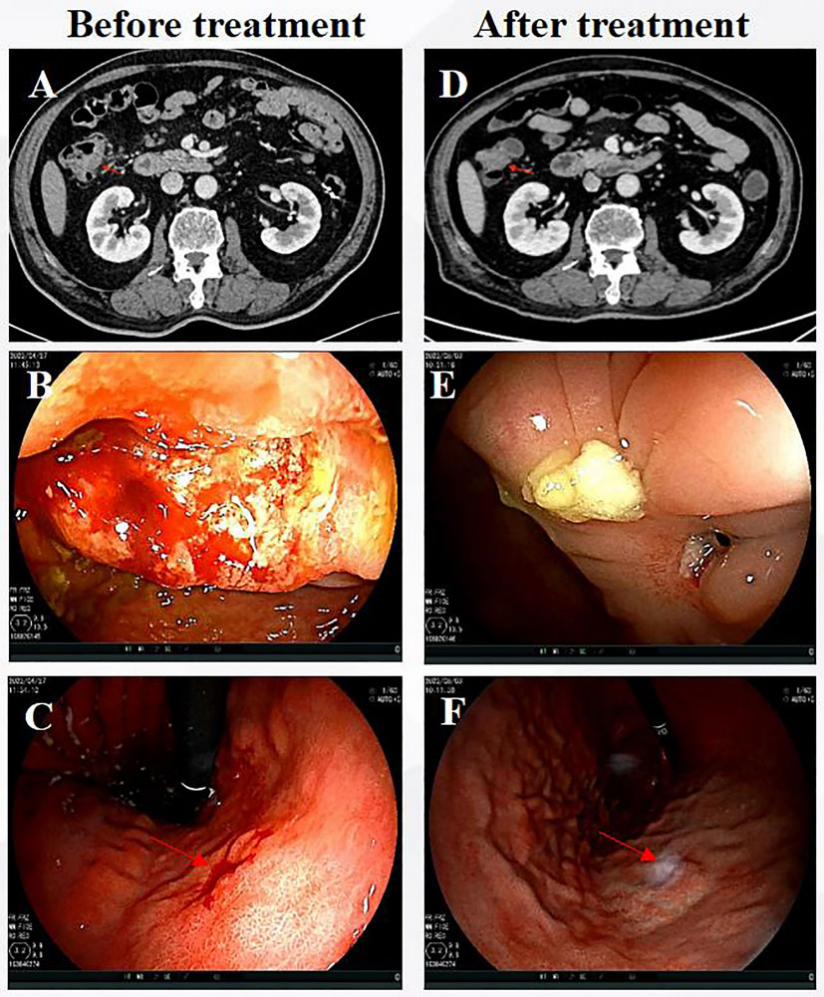
Imaging and endoscopic results before and after neoadjuvant treatment with trastuzumab, pertuzumab, and chemotherapy. (**A**) CT image of colon lesion before treatment, (**B**) colonoscopic image of colon cancer before treatment, (**C**) gastroscopic image of gastric cancer before treatment, (**D**) CT image of colon lesion after treatment, (**E**) colonoscopic image of colon cancer after treatment, and (**F**) gastroscopic image of gastric cancer after treatment.

After a comprehensive multidisciplinary discussion, the patient underwent four cycles of neoadjuvant FOLFOX6 (5 fluorouracil (5-FU)/leucovorin/oxaliplatin) combined with trastuzumab (450 mg) and pertuzumab (420 mg). Throughout the treatment period, the patient experienced only grade I nausea and vomiting, with no other documented adverse effects. Subsequent re-examination using whole abdominal CT revealed a noticeable reduction in size of the ascending colon lesion and surrounding lymph nodes compared to previous scans (Fig. 1D). Colonoscopy indicated scar-like changes without any detectable residual tumor cells in the hepatic flexure of the colon (pCR, Fig. 1E), while gastroscopy showed extensive chronic inflammation in the stomach body and severe glandular atrophy (pCR, Fig. 1F). One week later, laparoscopic right hemicolectomy and gastric endoscopic submucosal dissection were performed on the patient. According to the postoperative pathological report, both colon cancer and gastric cancer were staged as ypT0N0M0 (Fig. S2, see online supplementary material). Nine days after surgery, the patient recovered from pneumonia and was discharged from hospital. Subsequently, consolidation therapy using the same treatment regimen was continued for one cycle. A follow-up examination conducted 12 months after surgery showed no signs of recurrence.

Over the past two decades, significant advancements have been achieved in the management of colorectal cancer through extensive investigation into tumor-driving biomarkers. For advanced colon cancer patients with microsatellite instability-high, neoadjuvant immunotherapy has emerged as a standard therapeutic approach [5]. However, for most advanced cases, the neoadjuvant treatment strategy still relies on conventional fluoropyrimidine-based chemotherapy regimens such as FOLFOX and capecitabine/oxaliplatin (CAPOX), with limited pCR rates [6]. Therefore, it remains crucial to explore novel therapeutic biomarkers in order to enhance the rate of complete response and further improve the prognosis of patients with locally advanced colon cancer.

HER2-targeted therapy has shown remarkable activity against HER2-positive metastatic colorectal cancer in recent years [2]. Therefore, it is of significant clinical value to further investigate the efficacy of HER2-targeted therapy in the first-line metastatic, adjuvant, and neoadjuvant settings. In the present study, we reported a patient with locally advanced HER2-positive colon cancer who achieved pCR to four cycles of neoadjuvant chemotherapy combined with trastuzumab and pertuzumab, while experiencing only mild drug-related adverse events. After a postoperative follow-up time of 12 months, the patient remained alive without any signs of recurrence. According to these encouraging results, we believe that HER2-targeting treatment strategies hold great potential as a novel approach for treating HER2-positive colorectal cancer patients in the future.

Accurately detecting the expression level of HER2 in colorectal cancer is crucial for the anti-tumor therapy targeting HER2. In colorectal cancer, HER2 overexpression is traditionally evaluated by IHC and *in situ* hybridization [7]. In this study, the IHC analysis indicated the patient's HER2-negative status. Surprisingly, our NGS results revealed ERBB2 gene amplification in this patient, which influenced our decision to opt for HER2-targeted therapy. Previous study has demonstrated that despite a robust concordance between IHC and NGS in detecting HER2 expression, there still exists an 8% discrepancy [8]. The spatial heterogeneity of HER2 expression in tumor tissues suggests the importance of further assessing HER2 expression through NGS or other methods. In addition to providing copy number variation readings to quantify the extent of amplification, NGS can provide additional mutational information. In this study, the patient exhibited not only HER2 amplification but also an additional HER2 exon mutation. Mutations in ERBB2 are found in ∼5% of patients with colorectal cancer, and some of these mutations can co-occur with ERBB2 amplification. Studies have shown that ERBB2 gene mutations in colorectal cancer occur most frequently in exon 21, followed by exons 19 and 20 (as in this study) [9]. Although anti-HER2 therapy in patients with colorectal cancer harboring various ERBB2 gene mutations lacks sufficient evidence, some researchers think that recommending it as a treatment option is worth exploring [10].

Interestingly, apart from colon cancer, this patient also presented with early-stage gastric cancer. Considering the potential need for additional surgery following endoscopic submucosal dissection, we opted not to prioritize resecting the gastric cancer lesion in order to avoid multiple abdominal procedures. Surprisingly, subsequent to neoadjuvant chemotherapy combined with HER2-targeted therapy, complete disappearance of the patient's gastric cancer tumor cells was observed as well. In the absence of further confirmation regarding HER2 expression in gastric cancer, it remains uncertain whether the therapeutic effect primarily stems from chemotherapy, HER2-targeted therapy, or a combination thereof.

Our study demonstrates that neoadjuvant chemotherapy combined with trastuzumab and pertuzumab is an effective treatment strategy for locally advanced HER2-positive colon cancer patients, indicating the potential of HER2 targeting in neoadjuvant therapy and highlighting the importance of assessing HER2 status in all colorectal cancer patients. However, it should be noted that the sample size provided in this study was too small to conduct a statistical analysis, which limits the reliability and generalizability of our results. Therefore, more prospective studies are still needed to verify this issue. Additionally, enhancing the pCR rate of rectal cancer patients, particularly those with low rectal cancer, holds significant importance for preserving anal function. It is worth investigating whether the addition of dual anti-HER2 therapy to total neoadjuvant therapy could potentially augment the pCR rate in rectal cancer patients with HER2 amplification, or if these patients can be spared from radiotherapy within this treatment regimen without compromising efficacy.

## Supplementary Material

pbae033_Supplemental_File
