# Supplementary material for: Dual anti-HER2 therapy combined with chemotherapy as a novel neoadjuvant treatment option for locally advanced HER2-positive and microsatellite stable colon cancer
Source: Precis Clin Med. 2024 Dec 10;8(1):pbae033. doi: 10.1093/pcmedi/pbae033 (PMC11715517; doi:10.1093/pcmedi/pbae033)
Supplement: pbae033_Supplemental_File [file pbae033_supplemental_file.docx]

**Supplementary Figures**


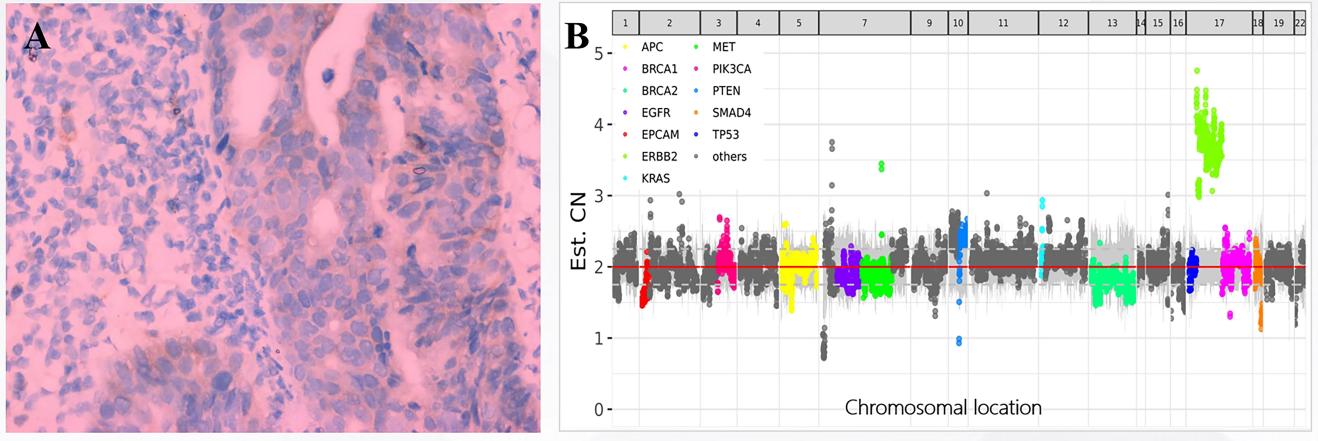


Figure S1. HER2 test results from colon cancer biopsy tissue. (A) Immunohistochemical examination of HER2 protein expression; (B) Genomic Copy Number Profile including ERBB2: The dots on the graph represent capture intervals for genes, with highly colored highlights indicating genes that focus on copy number variation. The horizontal axis indicates the chromosomal location of each gene, while the vertical axis represents the calculated copy number based on NGS methodology (the red horizontal line denotes the copy number of a normal gene).


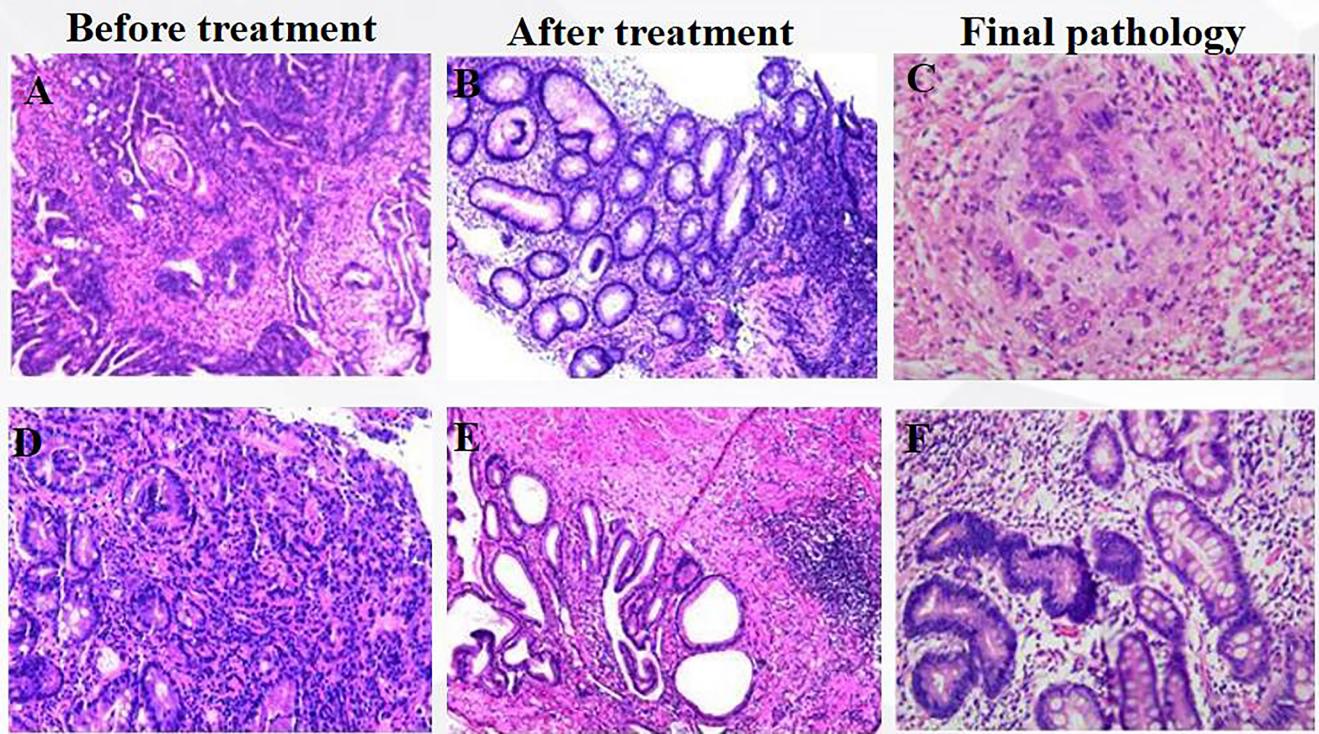


Figure S2. Histopathological images stained with hematoxylin and eosin. (A) Colon cancer tissue before treatment; (B) Colon cancer tissue after treatment; (C) Colon cancer tissue after final pathologic assessment; (D) Gastric cancer tissue before treatment; (E) Gastric cancer tissue after treatment; (F) Gastric cancer tissue after final pathologic assessment.
